# Screens, Blue Light, and Epigenetics: Unveiling the Hidden Impact on Skin Aging

**DOI:** 10.1093/asjof/ojae088

**Published:** 2024-10-09

**Authors:** Diala Haykal

I am writing to present a novel perspective on the emerging concern regarding blue light exposure from screens and its potential impact on skin aging, with a particular focus on the role of epigenetics. The widespread use of digital devices has led to increased exposure to blue light, which has become a significant point of interest in cosmetic dermatology. This letter aims to discuss the underlying mechanisms by which blue light contributes to skin aging and propose that epigenetic modifications could be a key factor in this process.

## BLUE LIGHT EXPOSURE AND SKIN HEALTH

Blue light, characterized by wavelengths between 400 and 490 nm, is a component of the visible light spectrum emitted by the sun, as well as by digital screens, light-emitting diode lights, and other artificial sources. Although ultraviolet (UV) radiation has been extensively studied for its harmful effects on the skin, including photoaging and carcinogenesis, the impact of blue light on skin health has only recently gained attention. Current research suggests that blue light penetrates the skin more deeply than UV radiation, potentially leading to oxidative stress, inflammation, and subsequent collagen degradation, all of which are hallmark processes of skin aging.^1^

## THE ROLE OF EPIGENETICS IN SKIN AGING

Epigenetics, the study of heritable changes in gene expression that do not involve alterations to the underlying DNA sequence, has revolutionized our understanding of how environmental factors can influence cellular function and contribute to aging.^2^ Epigenetic modifications, such as DNA methylation, histone modification, and changes in noncoding RNA expression, can regulate the expression of genes involved in key cellular processes, including those that maintain skin integrity and repair. Recent studies have demonstrated that environmental stressors, such as UV radiation, can induce epigenetic changes in skin cells, leading to altered gene expression profiles associated with premature aging. Given the similarities in the biological pathways affected by UV and blue light, it is plausible to hypothesize that blue light also induces epigenetic modifications that contribute to skin aging.

## HYPOTHESIS: BLUE LIGHT, EPIGENETICS, AND SKIN AGING

Chronic exposure to blue light from screens could lead to epigenetic alterations in skin cells, driving the aging process in a manner similar to UV-induced photodamage. This hypothesis is supported by the observation that oxidative stress, a known consequence of blue light exposure, is a potent inducer of epigenetic changes. For instance, oxidative stress can alter DNA methylation patterns and histone modifications, leading to dysregulation of genes involved in collagen synthesis, inflammatory responses, and cellular repair mechanisms. Over time, these epigenetic changes could accumulate, contributing to the visible signs of aging, such as wrinkles, loss of elasticity, and pigmentation disorders.^3^

## IMPLICATIONS FOR COSMETIC DERMATOLOGY

Understanding the epigenetic impact of blue light on the skin could have significant implications for the development of targeted skincare interventions. The cosmetic dermatology field is currently evolving in response to increasing evidence of environmental factors, such as blue light, that contribute to skin aging. If blue light is indeed shown to cause epigenetic changes that accelerate skin aging, this would justify the use of specialized products designed to protect against blue light exposure. Already, the market is seeing an influx of skincare products that claim to offer blue light protection, often incorporating ingredients like antioxidants, which are known to combat oxidative stress.

However, if the link between blue light and epigenetic modifications is conclusively established, future skincare products could be designed to specifically target and mitigate these epigenetic changes. For example, products that promote the repair of DNA methylation patterns or support histone modifications could emerge as a novel approach to antiaging skincare. Moreover, these findings could also influence recommendations for daily skincare routines, particularly for individuals who spend prolonged periods in front of screens. Dermatologists should begin to advise the use of blue light protective creams as a standard part of antiaging regimens, similar to sunscreens recommended to protect against UV damage.^4^ Additionally, cosmetic procedures evolve to address blue light–induced aging more directly. For instance, treatments that focus on reversing epigenetic alterations, such as certain types of laser therapy or topical applications, could become more prevalent. This could represent a new frontier in antiaging treatments, emphasizing not only on the visible effects of aging, but also the underlying molecular changes that contribute to these effects.^5^

In conclusion, although the research on blue light and skin aging is still in its infancy, the potential role of epigenetics in mediating these effects warrants further investigation. By exploring the intersection of blue light exposure, epigenetics, and skin aging, we uncover new insights that could transform our approach to skincare and aging prevention in the digital age.

## References

[1] Kumari J , Das K, Babaei M, Rokni GR, Goldust M. The impact of blue light and digital screens on the skin. J Cosmet Dermatol. 2023;22:1185–1190. doi: 10.1111/jocd.15576 36594795

[2] Krutmann J , Bouloc A, Sore G, Bernard BA, Passeron T. The skin aging exposome. J Dermatol Sci. 2017;85:152–161. doi: 10.1016/j.jdermsci.2016.09.015 27720464

[3] Coats JG , Maktabi B, Abou-Dahech MS, Baki G. Blue light protection, part I-effects of blue light on the skin. J Cosmet Dermatol. 2021;20:714–717. doi: 10.1111/jocd.13837 33247615

[4] Christensen T , Johnsen BJ, Bruzell EM. Violet-blue light exposure of the skin: is there need for protection? Photochem Photobiol Sci. 2021;20:615–625. doi: 10.1007/s43630-021-00043-9 33893982

[5] Campiche R , Curpen SJ, Lutchmanen-Kolanthan V, et al Pigmentation effects of blue light irradiation on skin and how to protect against them. Int J Cosmet Sci. 2020;42:399–406. doi: 10.1111/ics.12637 32478879 PMC7496068

